# Early Amyloid Formation and Neuroinflammatory Response in a Bigenic Mouse Model Expressing Human α‐Synuclein and Aβ

**DOI:** 10.1155/padi/7303965

**Published:** 2026-06-05

**Authors:** Radhika Thakore, Markus Aldén, Nadja Gustavsson, Lukas Danielson, Lívia Lins, Jorge Domínguez Sánchez, Ana Rosenthal Arensburg, Agnes Paulus, Iran Augusto Neves da Silva, Valeriia Skoryk, Rakez Kayed, Gunnar K. Gouras, Tomas Deierborg, Andreas Heuer, Sabine C. Konings, Oxana Klementieva

**Affiliations:** ^1^ Medical Microspectroscopy, Department of Experimental Medical Science, NanoLund, Multipark, Lund University, Lund, Sweden, lu.se; ^2^ Basal Ganglia Pathophysiology, Department of Experimental Medical Science, Lund University, Lund, Sweden, lu.se; ^3^ Behavioural Neuroscience Laboratory, Department of Experimental Medical Science, Lund University, Lund, Sweden, lu.se; ^4^ Neurophysiology Laboratory, Department of Physiology, Federal University of Sergipe, São Cristóvão, Brazil, ufs.br; ^5^ Department of Neurology and Mitchell Center for Neurodegenerative Diseases, University of Texas Medical Branch, Galveston, Texas, USA, utmb.edu; ^6^ Experimental Dementia Research Unit, Department of Experimental Medical Science, Lund University, Lund, Sweden, lu.se; ^7^ Experimental Neuroinflammation Laboratory, Department of Experimental Medical Science, Lund University, Lund, Sweden, lu.se; ^8^ Department of Molecular and Cellular Neurobiology, CNCR Center for Neurogenomics & Cognitive Research, Vrije Universiteit, Amsterdam, Netherlands, vu.nl

**Keywords:** α-synuclein, amyloid microdeposition, amyloid-βpeptides, microglia

## Abstract

Protein aggregation is a hallmark of several neurodegenerative diseases, including Alzheimer’s disease, Parkinson’s disease and dementia with Lewy bodies. A common feature of these disorders is the misfolding and aggregation of α‐synuclein (α‐syn) and amyloid‐β (Aβ) proteins into amyloid structures, which disrupt cellular homoeostasis and drive disease progression. While Aβ typically forms extracellular deposits and α‐syn accumulates in intracellular inclusions, both ultimately contribute to neuronal damage and neurodegeneration. Increasing in vitro evidence suggests that these proteins can interact, altering their structural properties and, in turn, their biological effects; however, the consequences of their co‐occurrence in vivo remain unclear. To address this gap, we examined whether α‐syn modulates Aβ deposition and associated neuroinflammation at early stages using a physiologically relevant bigenic mouse model coexpressing human α‐syn and APP knock‐in Aβ. Using combined histological and biochemical analysis, we characterised Aβ load and microglial responses at early time points. Our results indicate that α‐syn expression is associated with altered early Aβ deposition and microglial morphology in vivo. Specifically, while early Aβ deposits were detected in both Aβ/α‐syn and Aβ‐control mice from 2 months of age, at 4 and 6 months, reduced number and size of Aβ microdeposits was observed in the Aβ/α‐syn model. The reduction in Aβ load was accompanied by a more ramified microglial morphology consistent with a less activated microglial state. Whether this delayed response reflects protection or impaired immune surveillance remains unclear. Our findings highlight the complexity of indirect Aβ and α‐syn interactions and the need for further studies to clarify their functional impact. The newly generated bigenic mice provide a relevant platform to investigate early co‐pathology and its role in disease progression.

**Summary**
• Bigenic model (Aβ/α‐syn): Generated a mouse line combining APP triple knock‐in AD mutations with human α‐syn A53T to study interplay of Aβ and α‐syn, aggregation‐prone human proteins in vivo*.*
• Key findings: α‐syn attenuates early amyloid aggregation, Aβ/α‐syn mice displayed fewer and smaller Aβ deposits at 4 and 6 months compared to Aβ‐only mice. Importantly, microglia in Aβ/α‐syn mice preserved a more homoeostatic, complex morphology, suggesting changes in microglia morphology upon Aβ microdeposits in Aβ/α‐syn mouse model.

## 1. Introduction

Protein aggregation is a central feature of several neurodegenerative disorders, collectively termed amyloidopathies [1]. These include Alzheimer’s disease (AD), Parkinson’s disease (PD) and dementia with Lewy bodies (DLB), all of which are characterised by the misfolding of normally soluble proteins into amyloid structures [2] that disrupt cellular homoeostasis and drive neurodegeneration [3–5].

Two of the most extensively studied amyloidogenic proteins are amyloid‐β (Aβ) and α‐synuclein (α‐syn). Aβ is a peptide of 36–43 amino acids derived from the amyloid precursor protein (APP) and is the principal component of amyloid plaques in AD [6]. Aβ is generated from the APP through amyloidogenic cleavage by β‐ and *γ*‐secretases [7]. Aβ readily aggregates into oligomers and fibrils that accumulate extracellularly and are closely associated with synaptic dysfunction and neurotoxicity [8]. Aβ aggregation disrupts cellular functions, including protein degradation, axonal transport, autophagy and apoptosis [9]. Advanced stages of AD have been characterised by mature Aβ plaques [10], but smaller deposits emerge earlier in the disease course and are thought to represent initial amyloid pathology events [11–14]. Aβ accumulation activates inflammatory pathways, and the resulting inflammatory environment can further enhance amyloid aggregation and neuronal injury [15]. Understanding the contribution of amyloid microdeposits to neurodegeneration is important to identify cellular changes that precede irreversible damage.

Evidence suggests that Aβ does not act in isolation but can interact with other amyloidogenic proteins, including α‐syn, influencing their aggregation pathways and potentially exacerbating neurodegenerative processes [16]. α‐Syn is a presynaptic protein involved in the regulation of synaptic vesicles [17]. The aggregation of α‐syn is a key pathological feature of PD and has also been observed in AD [18, 19]. The misfolding and aggregation of α‐syn disrupt normal cellular functions, including neurotransmission and synaptic plasticity [20]. Posttranslational modifications of α‐syn, such as phosphorylation and ubiquitination, play significant roles in its aggregation and toxicity [21]. Moreover, α‐syn has been associated with amyloid plaques through the nonamyloid component (NAC), suggesting potential interactions between α‐syn and Aβ that may influence plaque formation and amyloid aggregation [19]. This raised the hypothesis that α‐syn expression could modulate early Aβ pathology. Since then, multiple in vitro studies have shown that α‐syn can interact with Aβ, acting as seeds that influence each other’s aggregation pathways; [22] α‐syn fibrils exert a stronger seeding effect on Aβ aggregation than vice versa, suggesting that cross‐seeding could contribute to the overlapping pathologies observed in AD and Lewy body disorders [23]. Molecular dynamic simulations further support a strong probability of association between α‐syn and Aβ, driven by electrostatic and hydrophobic interactions [24]. Preformed α‐syn oligomers have also been shown to act as templates for amyloid fibril growth, whereas monomers are restricted from nucleation in the presence of vesicles [22].

Copathology between Aβ and α‐syn has been extensively modelled also in vivo. Double‐transgenic studies demonstrated that Aβ can exacerbate α‐syn–related phenotypes: It was reported that the coexpression of Aβ and human α‐syn enhanced α‐syn accumulation and neuronal deficits compared to single‐transgenic mice [25]. Extending this concept, Clinton et al. demonstrated synergistic interactions among Aβ, tau and α‐syn in triple‐transgenic mice, leading to accelerated neuropathology and cognitive decline compared to single‐ or double‐pathology models [26]. Subsequent studies revealed bidirectional modulation. Kallhoff et al. reported that loss of α‐syn increased plaque burden at later stages in APP transgenic mice [27], while Khan et al. showed that α‐syn influences AD‐like phenotypes both in vivo and in primary neurons [28]. In contrast, Bachhuber et al. found that α‐syn reduced Aβ plaque formation in a double‐transgenic setting, indicating that α‐syn can suppress plaque deposition [29].

More recent work has emphasised aggregation dynamics and seeding mechanisms. Bassil et al. demonstrated that preexisting Aβ plaques promote α‐syn spreading and downstream pathology [30], and Lloyd et al. described reciprocal aggravation of Aβ and α‐syn pathologies in a prion‐type model [31]. Barnes et al. further showed that both soluble and insoluble α‐syn species can propagate amyloid pathology in vivo [32].

Together, these studies demonstrate that Aβ and α‐syn can interact in vivo; however, this relationship is highly context‐dependent, varying with model design and protein expression levels. To study Aβ and α‐syn interactions, we bred a bigenic mouse (Aβ/α‐syn) model that expresses two human amyloidogenic proteins carrying mutations known to accelerate amyloid pathology:

1. APP knock‐in mice harbouring the Swedish (KM670/671NL), Arctic (E22G) and Beyreuther/Iberian (I716F) mutations; these mutations enhance Aβ production and subsequent Aβ accumulation while preserving physiological APP expression [33].
2. Human α‐syn carrying the A53T mutation [34]. The A53T substitution, originally identified in familial PD, confers a heightened propensity for α‐syn aggregation in vivo*.* This A53T mouse transgenic model exhibits widespread α‐syn expression across cortical, hippocampal and brainstem regions at early ages, [32] with α‐syn inclusions appearing around 9 months in a caudal pattern beginning in the midbrain and extending to the cerebellum, brainstem and spinal cord [34].

While such combinations of mutations are unlikely to occur in humans, the bigenic Aβ/α‐syn model provides an opportunity to study how α‐syn may influence Aβ pathology in vivo.

To evaluate the biological properties of Aβ microdeposits, microglial morphology was used as a readout of neuroinflammatory activation. In the resting state, microglia cells exhibit a highly ramified morphology with long, complex processes [35, 36]. Upon activation such as encountering Aβ microdeposits, microglia cells undergo characteristic structural changes, including shortening and simplification of processes and enlargement of the cell body, resulting in a more compact phenotype associated with immune activation [37]. Previous work from our group showed that early‐life microglial priming by lipopolysaccharide attenuates plaque load in AD mice, illustrating that inflammatory context can influence amyloid deposition [38]. In this study, we hypothesised that α‐syn expression modulates Aβ deposition and associated microglial responses during the early stages of Aβ pathology. To test this, we combined histological and biochemical analyses to assess Aβ deposition and microglial morphology in Aβ/α‐syn and Aβ‐only mice at early ages.

## 2. Methods

### 2.1. Animal Housing

All procedures were conducted in accordance with the guidelines on experimental animal research approved by Malmö–Lund Ethical Committee for Animal Research in Sweden (#5.8.18–11908). The mice used in this study were of comparable ages across genotypes, and both male and female mice were included in all groups. The mice were kept on a 12‐h light and dark schedule with *ad libitum* access to food and water. Four mouse lines were used.

 
**Aβ mice:** Humanised APP knock‐in mice heterozygous for the Swedish, Beyreuther/Iberian and Arctic mutations in the APP gene. The human APP knock‐in mice used in this study were homozygous C57BL/6‐APP(NL‐G‐F) (Riken RBRC06344), kindly provided by Saido [33].
 
**α-syn mice:** The α‐syn transgenic mice used in this study were heterozygous B6.Cg‐Tg(Prnp‐SNCA∗A53T)23Mkle/J mice (Jackson Laboratory, stock no. 006823), expressing the human A53T‐mutated SNCA gene under the murine prion protein (Prnp) promoter [34].
 
**Aβ/α-syn mice:** A newly generated heterozygous bigenic mouse model was established by crossing homozygous Aβ mice with α‐syn mice
 
**Wild-type (WT) mice**: C57BL6/J were used as WT control.

All experimental animals were maintained on a C57BL/6J background.

### 2.2. Genotyping

The mice were genotyped using the PCRBIO Rapid Extract PCR Kit (PB10.24). DNA was extracted according to the manufacturer’s instructions. PCR was performed by incubation at 95°C for 5 min, followed by 32 cycles of denaturation at 95°C for 15 s, annealing at 60°C for 30 s and elongation at 72°C for 30 s.

The primers given in Table 1 were used.

**TABLE 1:** PCR primers used for genotyping α‐syn, APP and WT mice.

Mice	Froward primers	Reverse primer
α‐syn mice	(5′–3′): TCATGAAAGGACTTTCAAAGGC	CCTCCCCCAGCCTAGACC
WT mice	(5′–3′): CTAGGCCACAGAATTGAAAGATCT	GTAGGTGGAAATTCTAGCATCATCC
Aβ mice	(5′–3′): CTCCTTGTGGCTGGCGGTCACAC	CTATCG TGGACCGAGAATGGTCATG

2% agarose gel electrophoresis was performed to visualise the DNA bands from the PCR products GelRed (Biotium 41003). The APP primers produce a 670‐bp band for endogenous mouse APP and an 850‐bp band for the human APP transgene observed only in Aβ/α‐syn and Aβ‐only mice.

The α‐syn primers generate a 324‐bp band for mouse α‐syn and a 500‐bp band for the human α‐syn transgene observed only in Aβ/α‐syn and α‐syn‐only mice.

### 2.3. Brain Tissue Collection and Preparation

Mice were sedated with isoflurane and transcardially perfused with phosphate buffer (PB, pH 7.4). Brains were dissected and divided into hemispheres: The left was processed for immunohistochemistry, and the right was snap‐frozen in dry ice and stored at −80°C for biochemical analysis. The left hemisphere was postfixed in 4% PFA at 4°C for 24 h, cryoprotected in 15% sucrose for 24 h followed by 30% sucrose and stored at 4°C until sectioning. Coronal sections were cut on a freezing‐stage microtome (Leica SM2010R) in alternating series. Sections were stored in antifreeze solution (30% sucrose [CAS 57–50‐1], 30% ethylene glycol [CAS 107–21‐1] in 0.1 M·PB, pH 7.4) at −20°C until use.

### 2.4. DAB Immunohistochemistry

DAB immunohistochemistry was performed on tissue sections to detect Aβ and α‐syn. Sections were washed in potassium phosphate–buffered saline (KPB), quenched for 15 min in 10% methanol/3% hydrogen peroxide and blocked in 5% normal horse serum (NHS) with 0.25% Triton X‐100 in KPBS (TX‐KPBS). For Aβ detection, sections were incubated overnight at 4°C with mouse anti‐82E1 antibody (1:1000, IBL America, 10323) in 5% NHS/0.25% TX‐KPBS, followed by a biotinylated horse antimouse secondary antibody (1:200, Vector). Signal was visualised with the Vectastain Elite ABC kit and DAB (Vector SK‐4100). Sections were mounted on gelatin‐coated slides, air‐dried, dehydrated in ethanol/xylene and coverslipped with DPX.

For phosphorylated α‐syn (Ser129) detection (1:500, Abcam, ab51253), the same protocol was followed with the following modifications: blocking in 5% normal goat serum (NGS), secondary goat antirabbit antibody (1:200, Vector) and haematoxylin counterstaining. After DAB development and counterstaining, sections were dehydrated through ethanol, cleared in Histolab‐Clear and mounted with DPX.

### 2.5. Immunofluorescence

For fluorescent immunohistochemistry, sections were washed in phosphate‐buffered saline (PBS) for 5 min, followed by blocking in a buffer containing 1% bovine serum albumin (BSA), 0.4% Triton‐X and 3% NGS in PBS. Tissue sections incubated with primary antibodies (Table 2) were incubated overnight at 4°C.

**TABLE 2:** Primary antibodies.

Primary antibodies	Dilution	Target
Iba1 (Fujifilm, 019–19741)	1:1000	Microglia
82E1 (IBL America, 10,323)	1:200	Aβ (1–4 a.a.)
6E10 conjugated with Alexa Fluor 488 (BioLegend, 803,013)	1:400	Aβ (1–16 a.a.)
LAMP1 (Abcam, ab25245)	1:200	Dystrophic neurites
α‐syn 211 (SC‐12767)	1:1000	Human α‐syn
p‐S129 (ab51253)	1:100,000	α‐syn phosphorylated at serine 129

After washing with PBS, sections were incubated in the dark for 1 h with secondary antibodies (Table 3) diluted in blocking buffer (1% BSA and 3% NGS in PBS).

**TABLE 3:** Secondary antibodies.

Secondary antibodies	Dilution	Manufacturer
Alexa Fluor 546 goat antirabbit	1:500	Invitrogen, A11010
Alexa Fluor 488 goat antimouse	1:500	Invitrogen, A11029
Alexa Fluor 568 goat antimouse	1:500	Invitrogen, A11031
Alexa Fluor 568 goat antirat	1:500	Invitrogen, A11077
Alexa Fluor 647 goat antirabbit	1:500	Invitrogen, A21244

Following secondary antibody incubation, sections were washed and stained with DAPI at a dilution of 1: 10,000 (Sigma‐Aldrich, D9542) diluted in PBS. For sections processed for α‐syn immunolabeling, DAPI was applied at a dilution of 1:2000 (Sigma‐Aldrich, D9542). After staining, the sections were mounted with Dako Fluorescence Mounting Medium (Agilent, S3023) on glass slides.

### 2.6. Microscopy

Imaging of brain sections labelled with DAB was performed using a bright‐field VS120 virtual microscopy slide scanning system (Olympus, Tokyo, Japan) with an objective of 20x. Imaging of Iba1‐ and 82E1‐fluorescently labelled sections was performed using a CLS Operetta high‐content imaging system (PerkinElmer) with a 20 × air objective and Harmony 4.9 software. Z‐stack images were acquired, and for quantitative analysis, a single in‐focus optical section was selected from each stack and used for measurements.

Imaging of fluorescently labelled sections for dystrophic neurites (DNs) and Aβ was performed using a Stellaris DMI8 confocal microscope (Leica Microsystems), equipped with a 20x water immersion objective. Z‐stack images were acquired using LasX 5.1.0 software. For imaging, we used the following laser settings: DAPI was imaged with 6.39% laser power and 15.1 gain, Alexa 488 (6E10) with 16.69% laser power and 33 gain, Alexa 568 (LAMP1) with 13.37% laser power and 29.3 gain. Imaging of the α‐syn 211 and p‐S129 was performed with Leica DMi8, LAS X software, HC PL APO 20x/0.80, 16Bit.

### 2.7. Image Analysis

DAB‐positive images were analysed using ImageJ 1.53t. The number of Aβ microdeposits was manually quantified using the cell counter plugin in ImageJ. To analyse the size distribution of amyloid microdeposits across the brain, sections were selected at four rostro caudal levels. Using the Allen Mouse Brain Atlas (Allen Mouse Brain Atlas, mouse.brain‐map.org/experiment/show/71717640), we matched sections to the approximate bregma coordinates: 1.42, −0.555, −1.855 and −3.58 mm. At each bregma, 10–30 individual 82E1‐positive Aβ microdeposits were randomly selected and manually measured. ImageJ software equipped with a polygon selection tool was used to quantify the area of each amyloid aggregate. Notably, interconnected microdeposits were considered a single entity for measurement purposes, whereas distinct nonconnected microdeposits were measured individually. All raw Aβ microdeposit area measurements from each animal, across all selected bregma levels, were exported from ImageJ and used for statistical analysis of Aβ microdeposit size distribution.

To investigate regional variations in amyloid microdeposits, all brain slices labelled with the 82E1 antibody were manually superimposed on the corresponding coronal Allen Mouse Brain Atlas to identify anatomical regions. The amyloid burden was quantified manually within the defined brain regions. Subsequently, IrfanView Version 644.60 was employed to generate a heatmap visualisation, where colour intensity ranged from blue (regions with the lowest number of microdeposits) to red (regions with the highest number of microdeposits).

To analyse the differences in microglial response around Aβ microdeposits in the cortex, images from the CLS Operetta high‐content imaging system were used. Images were acquired from the cortex of three coronal brain sections per animal, matched across genotypes by bregma coordinates (0.86, −0.22 and −1.70 mm), based on the Allen Brain Atlas. All identifiable 82E1‐positive Aβ microdeposits within these cortical sections were included in the analysis. All visible Aβ microdeposits within the region of interest were analysed. The 82E1‐positive Aβ microdeposits were manually located, and Harmony 4.9 software (PerkinElmer) was used to locate the microglial morphologies (Iba1 channel) and count the number of cells (DAPI channel). The assessment of the number and morphological characteristics of microglia was assessed at different distances from the centre of the 82E1‐positive Aβ microdeposits. Three concentric regions were defined around each Aβ microdeposit: R1: 0–50; R2: 50–100; R3: 100–125 µm. R2 and R3 were defined as annular rings excluding the inner zones (e.g., R2 excludes 0–50 µm). Microglial enumeration and morphological analyses were conducted separately for each region. Morphological analyses were done using the ‘Find Neurites’ analysis in the Harmony 4.9 software. This analysis counted the tree area, length, number of segments and number of extremities of the microglial processes. Comparisons were made within the corresponding regions across genotypes (e.g., R1_Aβ vs. R1_Aβ/α‐syn). Microglia in close proximity to 82E1‐positive Aβ microdeposits were manually analysed. The 82E1‐positive Aβ microdeposits were manually identified, and the microglia were quantified within a ∼10 µm radius. Any microglial cell with at least one process contacting the Aβ microdeposit was considered as associated.

To analyse the DNs, 20x z‐stack images from the Stellaris DMI8 confocal microscope were used. Quantification of DNs was performed with ImageJ 1.53t on the maximum projection images. DNs were identified in the 568 channel and the image was converted to 8‐bit and thresholded in ImageJ using constant settings that selected the DNs. The image was then binarised, and the area covered by DN was measured using the analysing tool.

### 2.8. qRT‐PCR

Total RNA was extracted according to the manufacturer’s instructions using the RNeasy Mini Kit (QIAGEN). cDNA was synthesised from total RNA using the SuperScript VILO cDNA Synthesis Kit (Thermo Fisher Scientific, Cat. No. 11754–050). Quantitative PCR was performed using SYBR Green I Master (Roche) on a LightCycler 480 system (Roche). Gene expression levels were calculated using the ΔΔCt method and normalised to the housekeeping gene GAPDH as described [39]. Primer sequences are listed in Table 4.

**TABLE 4:** Primer sequences used for qRT‐PCR analysis.

Gene	GenBank accession	Primer	Sequence (5′–3′)	Length (nt)	Amplicon size (bp)	PrimerBank ID
HuAPP	NM_201414	F	TCTCGTTCCTGACAAGTGCAA	21	116	228008405c2
		R	GCAAGTTGGTACTCTTCTCACTG	23		

HuSNCA	NM_001146054	F	AAGAGGGTGTTCTCTATGTAGGC	23	106	225690601c1
		R	GCTCCTCCAACATTTGTCACTT	22		

MsGapdh	NM_008084	F	AGGTCGGTGTGAACGGATTTG	21	123	6679937a1
		R	TGTAGACCATGTAGTTGAGGTCA	23		

### 2.9. Dot Blot Analysis

The soluble fraction of brain homogenates was prepared in Tris‐buffered saline containing 0.1% Tween‐20 (TBS‐T) buffer. Total protein concentration was first determined using the BCA protein assay, and samples were normalised to equal protein concentration before dot blot analysis. Equal amounts of protein were then spotted onto nitrocellulose membranes and allowed to air dry. Membranes were blocked in 5% nonfat dry milk prepared in TBS‐T for 1 h at room temperature.

Membranes were incubated overnight at 4°C with the primary antibody diluted in blocking buffer, followed by washing three times in TBS‐T. The membranes were then incubated with the appropriate horseradish peroxidase (HRP)–conjugated secondary antibody for 1 h at room temperature.

After additional washes in TBS‐T, signals were detected using enhanced chemiluminescence (ECL) according to the manufacturer’s instructions. Membranes were imaged using a chemiluminescence imaging system, and dot intensities were quantified using ImageJ software. Signal intensity was expressed as relative intensity in arbitrary units (a.u.).

### 2.10. Statistical Analysis

All statistical analyses were performed using GraphPad Prism Version 9.4.1 (681) for Windows. The normality of the data distribution was assessed using the Shapiro–Wilk test, supplemented by visual inspection of Q–Q plots. For datasets with three or more independent groups and normally distributed data, two‐way ANOVA was performed, with factors corresponding to genotype and distance from Aβ microdeposit (0–50, 50–100, and 100–125 μm). If significant main effects or interactions were found, post hoc comparisons were conducted using Tukey’s multiple comparisons test. For comparisons between two independent groups, unpaired two‐tailed *t*‐tests were used for normally distributed data. When data were not normally distributed, nonparametric tests were applied: Mann–Whitney *U test* for two‐group comparisons and the Kruskal–Wallis test for comparisons involving three or more groups. The Kruskal–Wallis test was used as a nonparametric alternative to one‐way ANOVA. When appropriate, Dunn’s multiple comparisons test with Bonferroni correction was applied following the Kruskal–Wallis test.

All statistical tests were two‐tailed, and significance was set at *p* ≤ 0.05. Data are presented as mean ± standard deviation (SD) for parametric datasets or median with interquartile range for nonparametric data. The experimental unit was the individual animal, *n* denotes the number of animals per group and N refers to the total number of analysed slices per group.

## 3. Results

### 3.1. Aβ and α‐Syn Accumulation in the Cortex of Aβ/α‐Syn Mice

One of our objectives was to validate that the newly developed Aβ/α‐syn mouse model carries the transgenes present in both APP and α‐syn parental lines. PCR analysis confirmed this expression: The 670‐bp band corresponds to endogenous mouse APP, whereas the 850‐bp band represents human APP (Figure 1(a)). Next, to validate the model, we performed double immunolabeling of brain sections from 9‐month‐old Aβ and Aβ/α‐syn mice using the N‐terminal–specific 82E1 anti‐Aβ antibody and an anti–α‐syn antibody. Both Aβ‐only and Aβ/α‐syn mice displayed Aβ microdeposits (Figure 1(b)), whereas WT animals, included as healthy controls, showed no deposits. The 9‐month time point was chosen as it represents the earliest stage at which α‐syn aggregation becomes detectable in α‐syn mice [34].

FIGURE 1Validation of Aβ and α‐syn in the Aβ/α‐syn mouse model. (a) Gel image showing polymerase chain reaction (PCR) amplified gene products. A band at 670 bp corresponding to the endogenous mouse APP gene was detected in WT, Aβ‐only and Aβ/α‐syn mice. The band at 850 bp corresponding to the human APP transgene was detected only in Aβ‐only and Aβ/α‐syn mice. (b) Representative images of DAB labelling at 9‐month‐old WT, Aβ‐only and Aβ/α‐syn brain sections labelled with the 82E1 antibody against N‐terminal Aβ. Insets show typical Aβ microdeposits that have been observed in Aβ/α‐syn and Aβ‐only mice, which are absent in WT mice. (c) Gel image showing PCR amplified gene products. A band at 324 bp corresponding to the endogenous mouse α‐syn gene was detected in WT, Aβ/α‐syn and α‐syn‐only mice. The band at 500 bp corresponding to the human α‐syn transgene was detected in Aβ/α‐syn and α‐syn–only mice. (d) Representative images of DAB labelling at 9‐month‐old WT, α‐syn‐only and Aβ/α‐syn mouse brain sections from the cortex labelled with antibody pS129 against phosphorylated α‐syn at serine residue 129.

**Figure:**
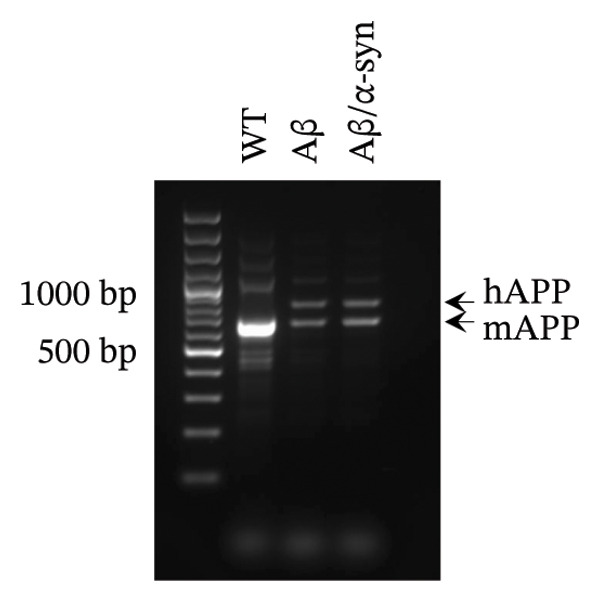
(a)

**Figure:**
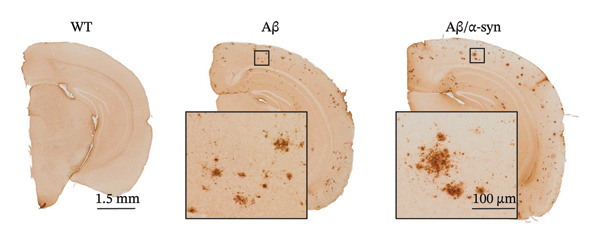
(b)

**Figure:**
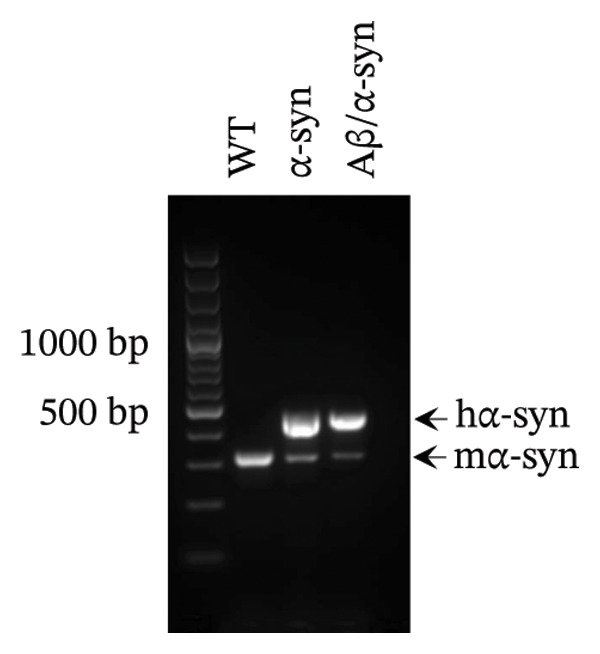
(c)

**Figure:**
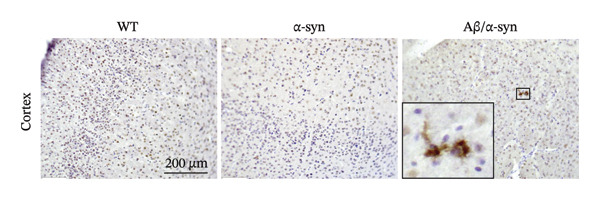
(d)

To verify the presence of the α‐syn transgene in Aβ/α‐syn mice, PCR analysis was performed, revealing a 500‐bp band identical to that observed in α‐syn mice (Figure 1(c)). Since phosphorylation at serine 129 (pS129) is a marker of α‐syn aggregation [40], we next examined its presence in the Aβ/α‐syn model. Immunohistochemistry using a pS129‐specific antibody confirmed α‐syn phosphorylation in the newly generated Aβ/α‐syn mice. At 9 months of age, phosphorylated α‐syn was detected in the cortex (Figure 1(d)), indicating that both Aβ and α‐syn were expressed in the same brain region and could therefore interact. Interestingly, in α‐syn mice, α‐syn aggregation at this age was restricted to the midbrain, cerebellum, brainstem and spinal cord, whereas in Aβ/α‐syn mice, cortical accumulation of phosphorylated α‐syn was clearly evident. Thus, we confirmed that the newly developed Aβ/α‐syn mouse model successfully exhibits both human Aβ and α‐syn within the same brain region, resulting in expression of both Aβ and α‐syn in the same cortical region.

### 3.2. Aβ/α‐Syn Mice Have Less Amyloid Microdeposits at 4 and 6 months

After confirming that Aβ/α‐syn mice express both human Aβ and α‐syn, we next examined early Aβ deposition and whether the presence of human α‐syn influences this process. Immunohistochemical labelling with the N‐terminal Aβ antibody 82E1 was performed on brain sections from Aβ‐only and Aβ/α‐syn mice at 2–4 months of age. Aβ microdeposits were defined as discrete 82E1‐positive spots clearly distinguishable from background staining and separated from neighbouring aggregates. The number of Aβ deposits and their cumulative area were quantified within matched cortical regions (Supporting Figures S1 and S2). Images were acquired under identical exposure settings and analysed using the same parameters in a blinded manner. The 82E1‐positive microdeposits detected at early time points were distinct from mature plaques, being smaller and morphologically diverse.

Specifically, these microdeposits exhibited a range of morphologies, including coreless, core‐containing, granular, projecting and irregular forms (Supporting Figure S1). At 2 and 3 months, Aβ microdeposit numbers did not differ significantly between genotypes (*p* = 0.70, *p* = 0.32; Supporting Figure S3). By 4 months, however, quantification showed that Aβ/α‐syn microdeposits were fewer (*p* = 0.03, Figure 2(a)) and smaller (*p* = 0.03, Figure 2(b)) than those in Aβ‐only controls at 4 months. The regional distribution of Aβ aggregates reveals widespread deposition in Aβ mice and a reduced, more restricted pattern in Aβ/α‐syn mice. Quantification of Aβ distribution demonstrates a lower aggregate burden in Aβ/α‐syn mice across multiple brain regions, with the most pronounced reduction in somatosensory and somatomotor cortices, indicating attenuation and redistribution of Aβ pathology in the presence of α‐syn (Figures 2(c), 2(d) and 2(e)).

FIGURE 2Aβ/α‐syn mice have less amyloid microdeposits at 4 months (a) Representative whole‐brain section and regional magnification of amyloid microdeposits. (b) Quantification of the number of amyloid microdeposits normalised per slice, mean ± SD, *n* = 6 animals per genotype; each black dot represents one animal, a two‐tailed unpaired *t*‐test, t (10) = 2.49, *p* = 0.0319. (c) Size distribution of the amyloid microdeposits in brain sections from Aβ‐only and Aβ/α‐syn mice (from 4 bregma points ∼ 1.42, −0.555, −1.855 and −3.58 mm, *n* = 6 animals per genotype. Statistics: a two tailed Mann–Whitney U test, *U* = 21,189, *p* = 0.0302. Median values are shown with a central line; quartiles are indicated with lower and upper lines. The number of slices analysed per animal was APP: *N* = 236, Aβ/α‐syn: *N* = 204, mean ± SD, Aβ‐only: 39.3 ± 1.6 and Aβ/α‐syn: 34.0 ± 9.6. The average Aβ microaggregate size per animal. Statistics: a two tailed Mann–Whitney *U* test, *U* = 7, *p* = 0.0931. (d) Regional distribution of amyloid microdeposits in Aβ‐only and Aβ/α‐syn mice. Frequencies represent the (%) of total microdeposits found in each anatomical region across four bregma intervals (∼1.42, −0.555, −1.855 and −3.58 mm) based on overlays with the Allen Mouse Brain Atlas. The heatmap shows aggregate frequency using a blue‐to‐red gradient, where blue indicates regions with low aggregate frequency and red indicates high microdeposits frequency. Grey areas denote regions where no microdeposits were detected. (e) Post hoc Dunn’s multiple comparisons test with Bonferroni correction, *p* > 0.05 for all comparisons.

**Figure:**
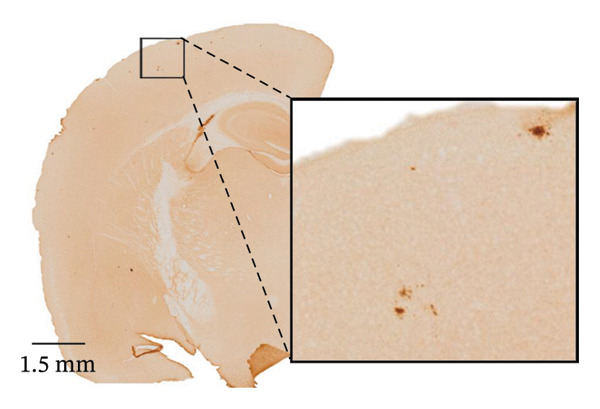
(a)

**Figure:**
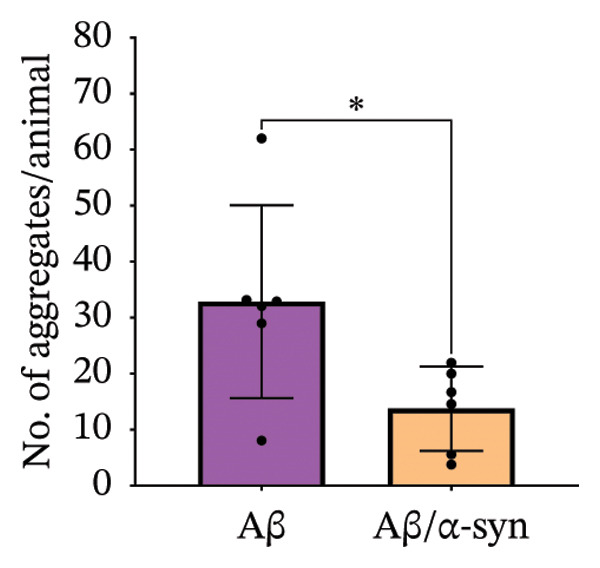
(b)

**Figure:**
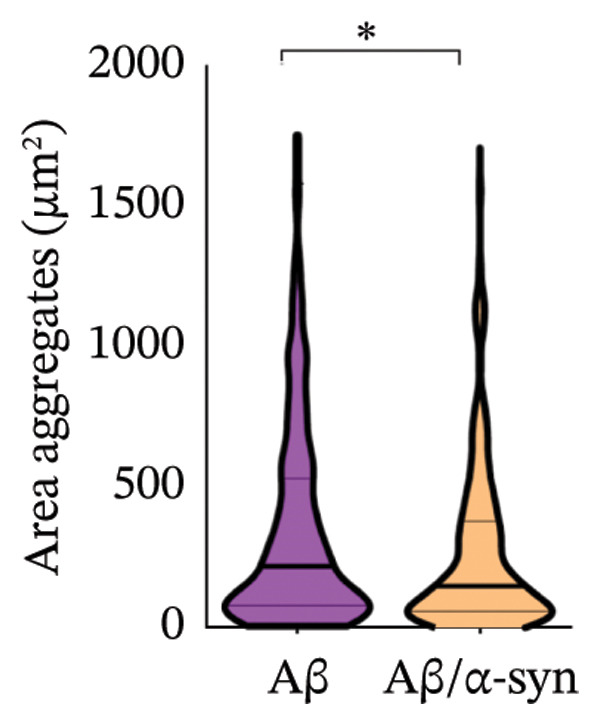
(c)

**Figure:**
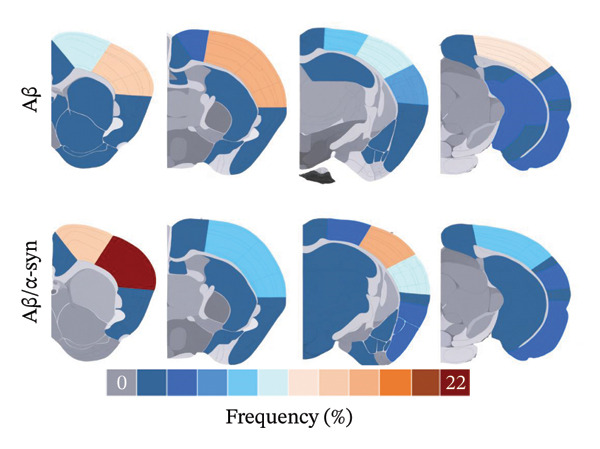
(d)

**Figure:**
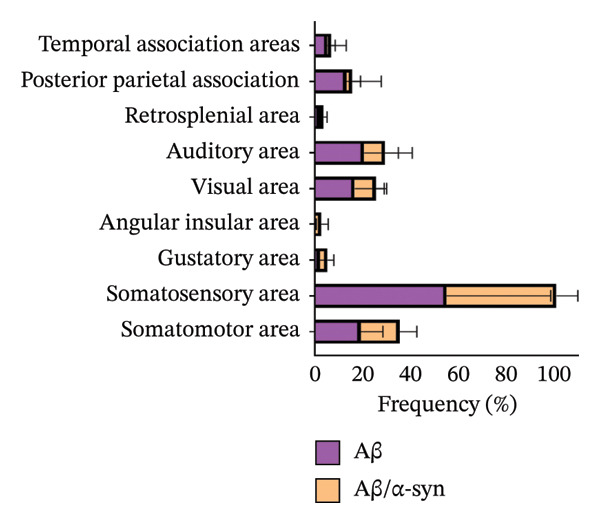
(e)

**FIGURE 4:**
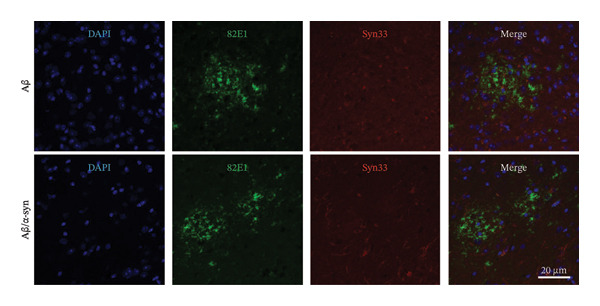
Limited cortical α‐syn aggregation at 6 months in Aβ/α‐syn mice. Representative immunofluorescence images of cortical sections from Aβ‐only and Aβ/α‐syn mice at 6 months of age. Nuclei are labelled with DAPI (blue). Aβ deposits are detected using the 82E1 antibody (green). Aggregated α‐syn is visualised using the aggregation‐specific Syn33 antibody (red). White arrows show Syn33 positive neurites. Upper and lower panels show representative fields containing 82E1‐positive Aβ microdeposits.

A similar trend was observed at 6 months. Aβ/α‐syn mice showed a marked reduction in both the number and size of amyloid microdeposits relative to Aβ‐only animals (Figure 3). To determine whether these differences might be associated with α‐syn pathology, we next examined cortical α‐syn aggregation. Immunostaining with phosphorylation‐specific antibodies against pS129 did not reveal aggregation‐associated signal at 4 months (Supporting Figure S5). At 6 months, analysis with the aggregation‐specific Syn33 antibody detected only sparse aggregated α‐syn signal in the cortex, with little or no colocalisation with 82E1‐positive Aβ deposits (Figure 4).

FIGURE 3Reduced amyloid aggregate burden in Aβ/α‐syn mice at 6 months. (a) Representative images of amyloid staining in coronal brain sections. Left: whole‐section overview; right: higher‐magnification inset of the boxed region. (b) Quantification of the number of amyloid microdeposits per slice at 6 months. Data are presented as median (IQR), *n* = 5‐6 animals per genotype; each black dot represents one animal. Statistics: a two‐tailed exact Mann–Whitney U test, *U* = 2, *p* = 0.00173). (c) Size distribution of amyloid microdeposits in brain sections from Aβ‐only and Aβ/α‐syn mice (four bregma levels: ∼1.42, −0.555, −1.855 and −3.58 mm). *n* = 5–6 animals per genotype. Statistics: a two‐tailed unpaired *t*‐test, *p* = 0.0221.

**Figure:**
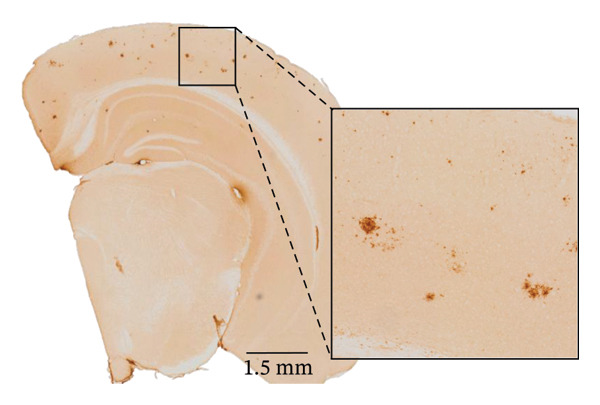
(a)

**Figure:**
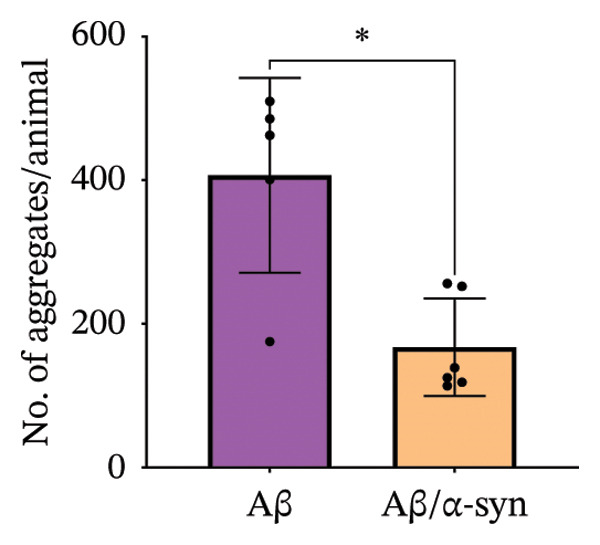
(b)

**Figure:**
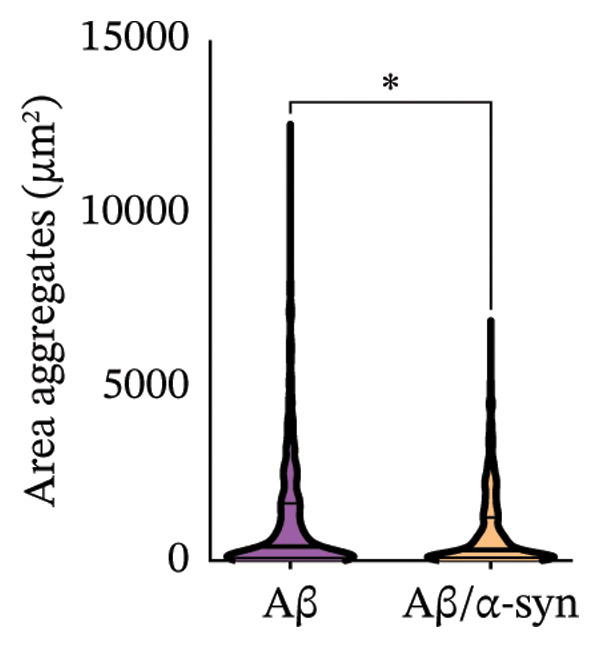
(c)

To verify that α‐syn was nevertheless expressed in the cortex at these ages, we performed dot blot analysis and qPCR. Dot blot analysis using a human‐specific α‐syn antibody confirmed cortical expression of the α‐syn protein at 4 and 6 months (Supporting Figure S4A,B), and qPCR detected human SNCA transcripts in Aβ/α‐syn brain tissue. In addition, human APP mRNA levels were comparable between Aβ‐only and Aβ/α‐syn mice (Supporting Figure S5C,D). Quantification of soluble Aβ fractions in nonmembrane and membrane‐associated protein extracts at 2–4 months revealed no significant differences between genotypes (Supporting Figure S6), indicating that the reduced amyloid burden observed in Aβ/α‐syn mice is unlikely to result from altered APP expression or Aβ production.

### 3.3. Presence of DNs in Aβ‐Only and Aβ/α‐Syn Mice

Next, we examined the presence of dystrophic neuronal processes, such as swollen axons or dendrites, so called DNs, which are closely associated with amyloid plaques [41] and frequently observed in periplaque regions and represent a characteristic feature of plaque‐associated neuronal damage [42–44].

To investigate the association between DNs and amyloid microdeposits, we performed immunofluorescence labelling using the lysosome‐associated membrane protein 1 (LAMP1) along with the N‐terminal Aβ antibody 6E10 to detect DN and Aβ, respectively [44]. We selected 4 months for analysis, as amyloid pathology was minimal at 2 and 3 months. At this age, LAMP1‐positive DNs were observed surrounding 6E10‐positive amyloid microdeposits (Figure 5(a)). Although the DN area in Aβ‐only mice appeared slightly larger than in Aβ/α‐syn mice, the difference did not reach statistical significance (Figure 5(b), *p* = 0.0817). Thus, Aβ expression alone does not lead to a significant increase in DN pathology.

FIGURE 5Dystrophic neurites around amyloid deposits. (a) Representative images of double immunofluorescence‐labelled brain sections with the LAMP1 antibody against dystrophic neurites (DNs) and the 6E10 antibody against N‐terminal Aβ. The images show DNs associated with 6E10 positive Aβ microdeposits for Aβ‐only and Aβ/α‐syn mice at 4 months (b) Quantification of DN area for Aβ‐only mice as compared to Aβ/α‐syn mice at 4 months. Statistics: *n* = 5–6 animals per genotype, each black dot represents one animal, Welch’s *t*‐test, no significance.

**Figure:**
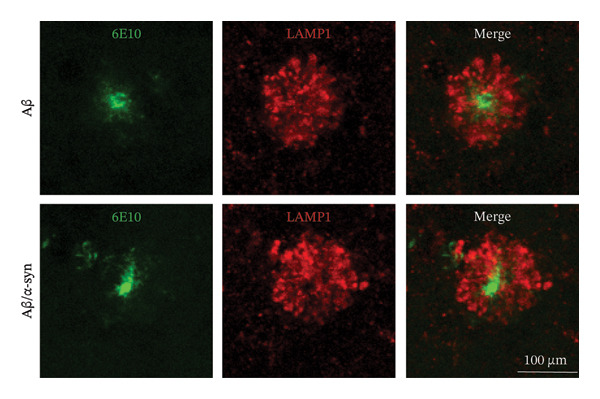
(a)

**Figure:**
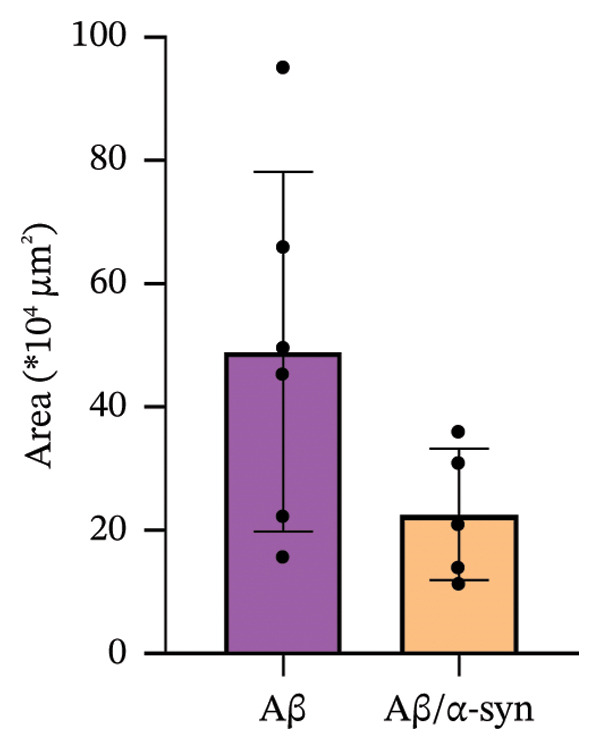
(b)

### 3.4. Altered Microglial Response in Aβ/α‐Syn Mice

Next, we examined microglia‐mediated inflammation, as it is a hallmark of many neurodegenerative diseases [45]. For example, microglial cells are activated following the deposition of Aβ fibrils, which is generally considered a triggering factor in the early steps of AD onset [46].

Immunofluorescence labelling was performed using the N‐terminal Aβ antibody 82E1 and the microglial marker Iba1 in 4‐month‐old Aβ and Aβ/α‐syn mice. Microglial responses were quantified at three distances from the centre of 82E1‐positive Aβ microdeposits: R1 (0–50), R2 (50–100) and R3 (100–125 µm), as well as in microglia directly associated with the microdeposits (Figure 6(a)). No significant differences were observed in the number of microglia associated with 82E1‐positive Aβ microdeposits between Aβ and Aβ/α‐syn mice (Figure 6(b), *p* = 0.6901), nor in the spatial distribution of microglia at distances R1–R3 from the microdeposits (Figure 6(c)). However, microglial morphology differed between genotypes, specifically, two‐way ANOVA showed no significant effect of distance or genotype × distance interaction. In contrast, genotype significantly affected several morphological parameters, including total process length (*p* = 0.0002), number of segments (*p* < 0.0001) and number of extremities per cell (*p* = 0.0001), indicating reduced process complexity in Aβ mice compared with Aβ/α‐syn mice (Figures 6(d), 6(e), 6(f), 6(g) and 6(h)). At 6 months, morphological analysis revealed differences in microglial structural parameters between genotypes. Microglia in Aβ mice exhibited reduced total process tree area, shorter total process length and fewer segments and extremities compared with Aβ/α‐syn mice, consistent with a more compact and less ramified morphology. In contrast, microglia in Aβ/α‐syn mice maintained a more complex, ramified morphology with greater process extension and branching (Figures 6(i), 6(j), 6(k), 6(l), 6(m), 6(n) and 6(o)). Two‐way ANOVA revealed a significant effect of genotype for all morphological parameters (*p* < 0.0001), whereas neither distance from the deposit nor the interaction between distance and genotype reached statistical significance.

FIGURE 6Morphology of microglia in proximity to 82E1‐positive Aβ microdeposits. (a) Schematic representation of the approach used to quantify microglial changes at three distances from the Aβ microdeposit: R1 (0–50), R2 (50–100) and R3 (100–125 µm), as well as Aβ‐associated microglia. (b) Quantification of the number of microglial cells associated with 82E1‐positive Aβ microdeposits (two‐tailed unpaired *t*‐test). (c) Quantification of the number of microglia immunolabelled by the Iba1 antibody in R1–R3 areas as indicated in panel A. Two‐way ANOVA, distance effect: *p* < 0.0001, genotype effect: *p* = 0.1235. (d) Schematic illustration of the different microglial morphologies analysed. Numbers indicate (1) total process tree area, (2) number of segments and (3) number of extremities. Quantification of (e) total process tree area (two‐way ANOVA: *p* (R2) = 0.0154, (f) total process length *p*(R2) = 0.0152, (g) number of segments *p* (R2) = 0.0274 and (h) number of extremities per cell *p*(R2) = 0.0147. (e–h) Quantification of microglial morphological parameters (total process tree area, total process length, number of segments and number of extremities). (i) Overview of tissue labelled with Iba1. (j) Representative immunofluorescence images showing Aβ deposits and surrounding microglia in Aβ‐only and Aβ/α‐syn mice. Scale bar: 50 µm. (k) Quantification of microglial cells located in plaque‐proximal regions (R1–R3). (l–o) Quantification of microglial morphology parameters at 6 months: (l) total process tree area, (m) total process length, (n) number of segments and (o) number of extremities per cell in regions R1–R3. Microglia in Aβ/α‐syn mice display increased process complexity compared with Aβ‐only mice. Data are presented as mean ± SD; each dot represents one animal (*n* = 5–6 animals per genotype). Statistical significance was determined by two‐way ANOVA followed by post hoc multiple comparison tests where indicated (^∗∗∗∗^ *p* < 0.0001).

**Figure:**
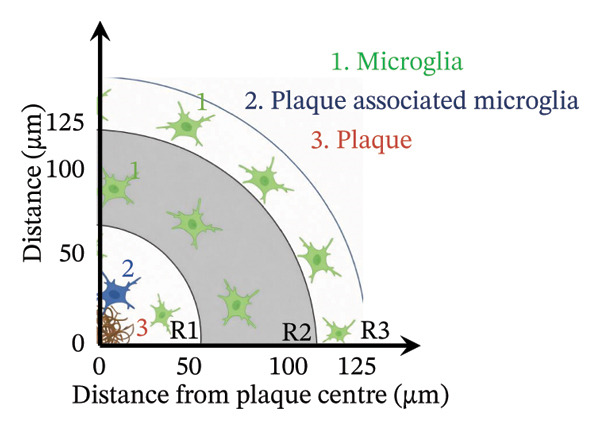
(a)

**Figure:**
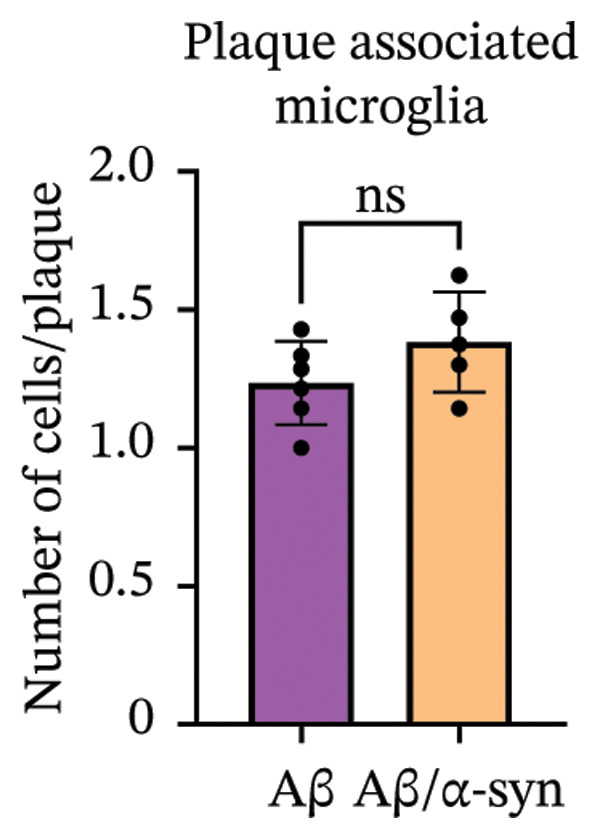
(b)

**Figure:**
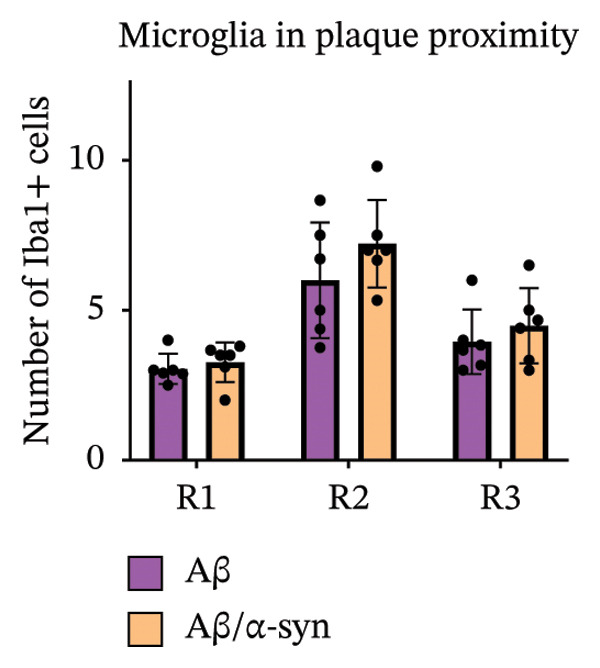
(c)

**Figure:**
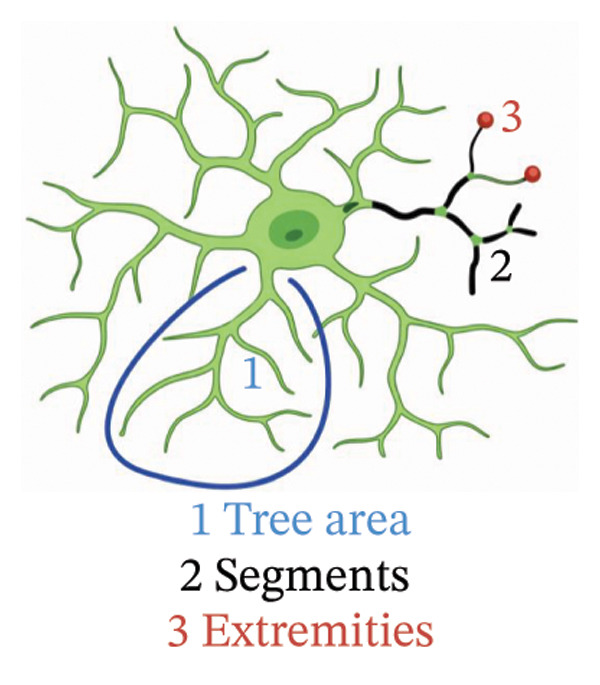
(d)

**Figure:**
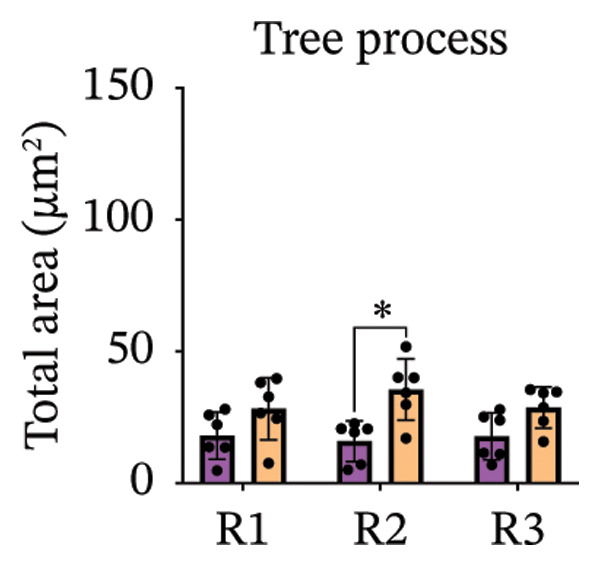
(e)

**Figure:**
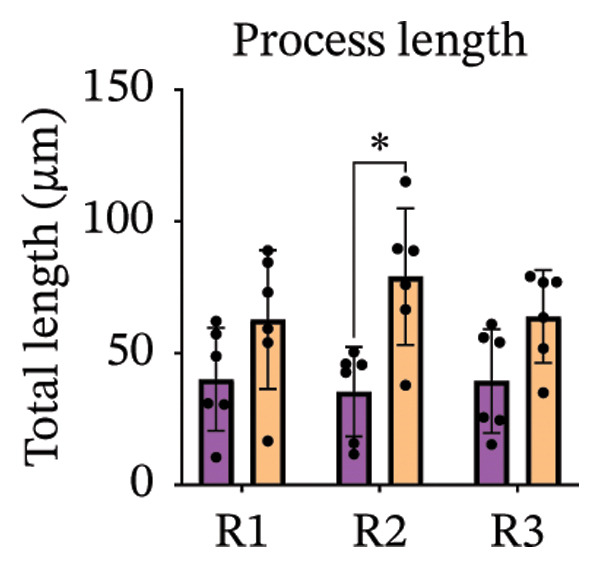
(f)

**Figure:**
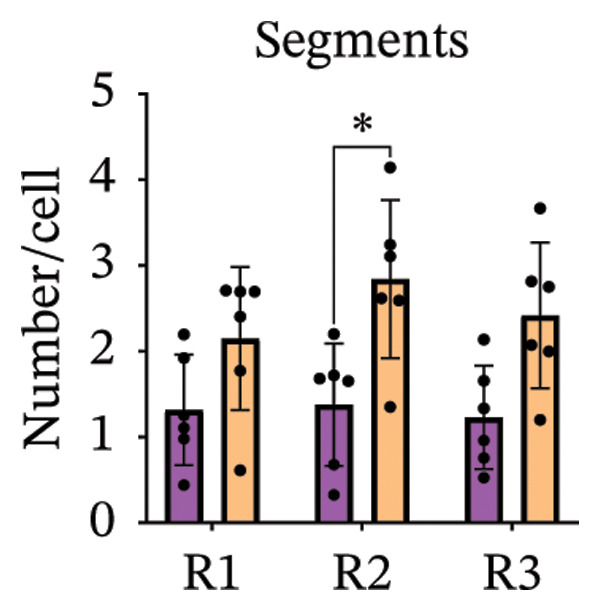
(g)

**Figure:**
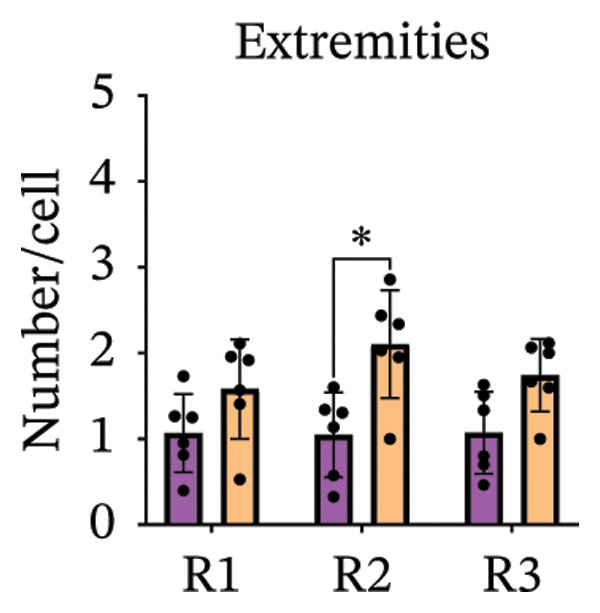
(h)

**Figure:**
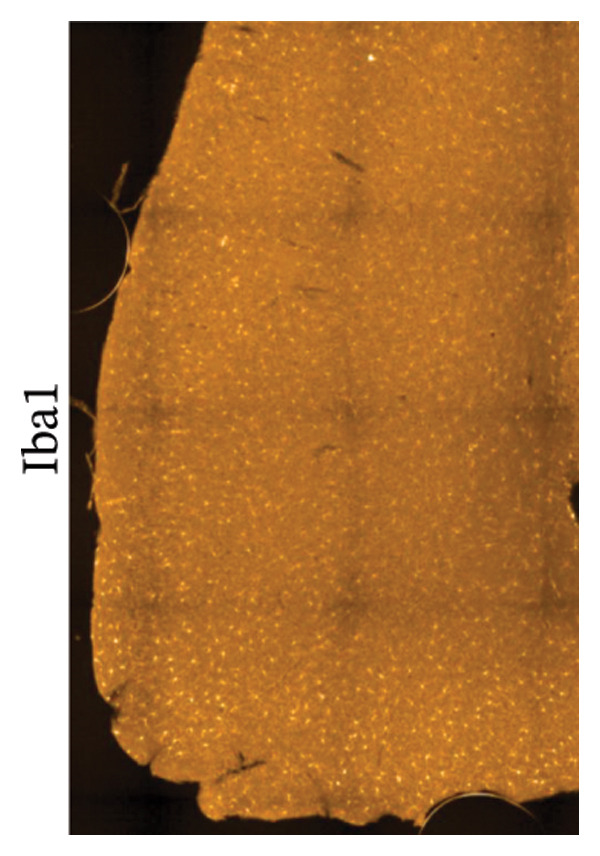
(i)

**Figure:**
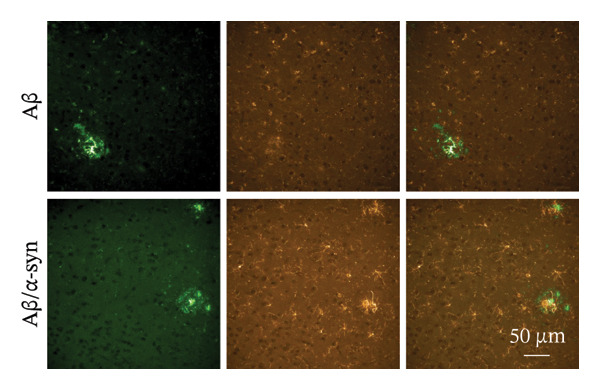
(j)

**Figure:**
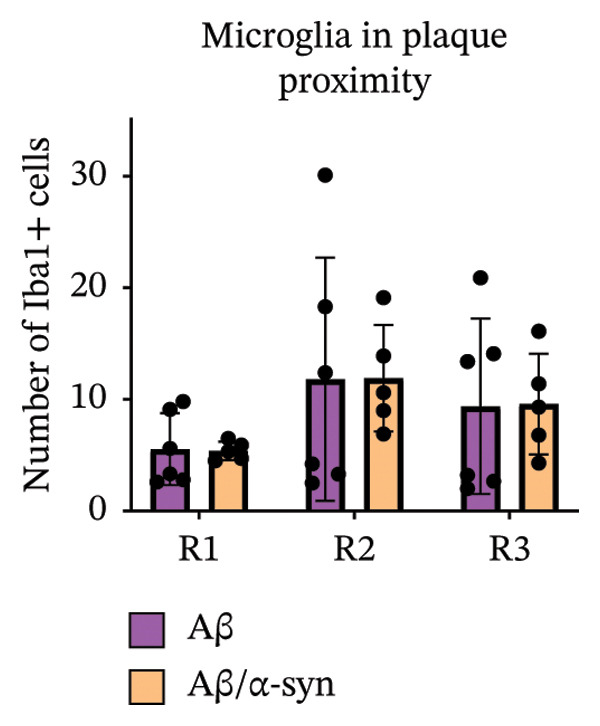
(k)

**Figure:**
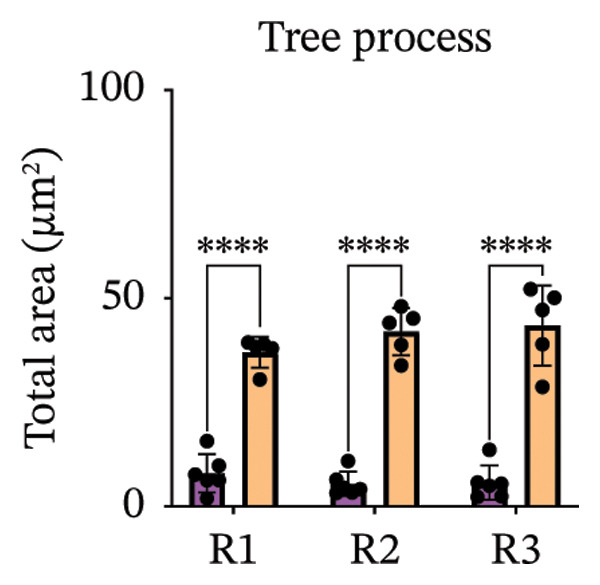
(l)

**Figure:**
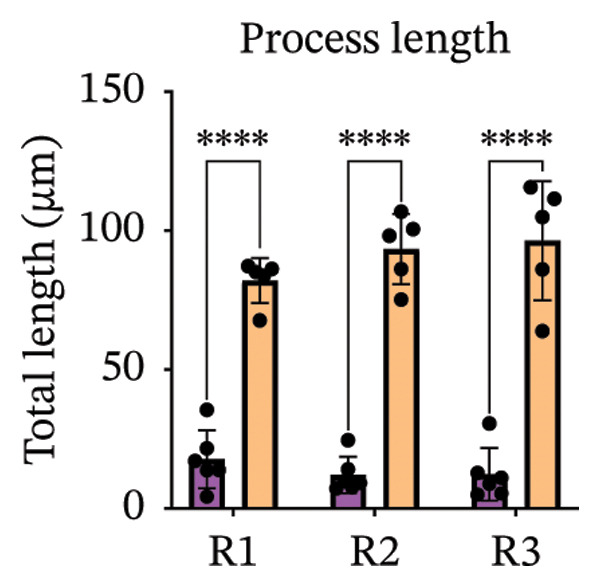
(m)

**Figure:**
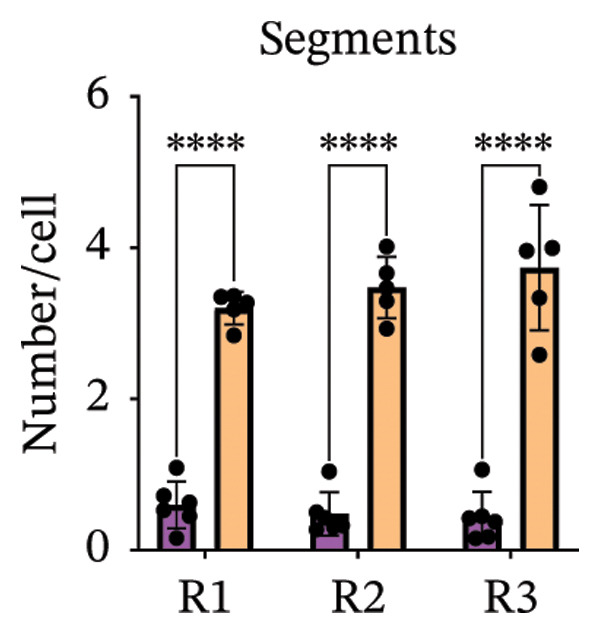
(n)

**Figure:**
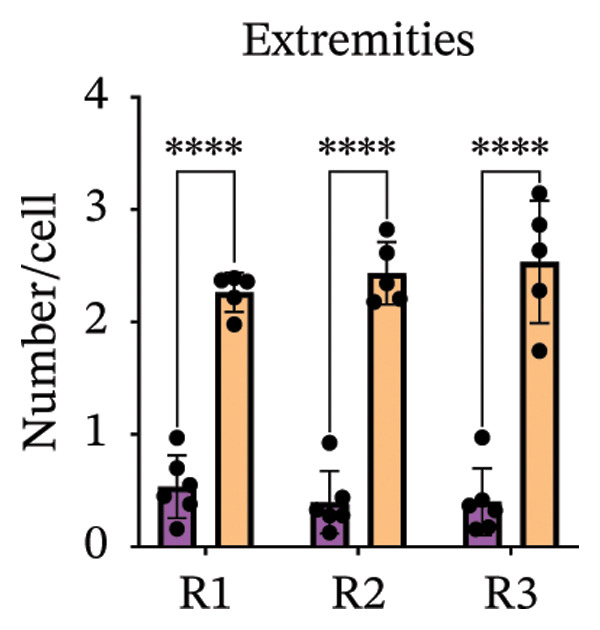
(o)

## 4. Discussion

Aβ plaques and Lewy bodies enriched in α‐syn represent the defining neuropathological features of AD and PDs, respectively. Considerable overlap between these pathologies has been documented: In DLB and in more than half of AD cases, α‐syn accumulates not only within Lewy bodies but also in DNs associated with Aβ plaques [47, 48]. Importantly, patients with copathology of aggregated Aβ and α‐syn exhibit reduced plaque load but experience faster disease progression than patients with pure AD [49–53]. These observations suggest that α‐syn may influence disease progression beyond simply increasing plaque load. Consistent with this, experimental evidence further indicates that Aβ and α‐syn can physically interact and coaggregate, [54, 55] yet the precise role of α‐syn in amyloid plaque formation remains unresolved. Recent neuropathological analyses further indicate that their relationship may extend beyond direct colocalisation, as increased α‐syn burden in the amygdala has been associated with elevated cortical Aβ deposition despite no corresponding increase in Aβ within the amygdala itself, suggesting a nonlocal and potentially more complex interplay between these pathologies [18].

In this study, we hypothesised that α‐syn influences early Aβ aggregation dynamics in vivo. To test this, we generated a bigenic line coexpressing human aggregation‐prone Aβ and α‐syn and examined early amyloid accumulation together with associated neuroinflammatory responses. In this model, α‐syn aggregation becomes detectable at 9 months, whereas at the earlier time points analysed in this study (4–6 months), no cortical aggregation was detected. Immunolabeling for total α‐syn (αS211) and phosphorylated α‐syn (pS129) did not reveal aggregation‐associated structures, and staining with the aggregation‐specific Syn33 antibody showed only minimal signal. Importantly, α‐syn expression levels were comparable across animals, indicating that the observed effects are unlikely to be attributable to differences in transgene expression. Likewise, soluble Aβ fractions were similar between genotypes, arguing against major changes in peptide production or clearance at these early stages. Despite the absence of detectable α‐syn aggregation or direct evidence of Aβ/α‐syn coaggregation, the bigenic mice exhibited a reduction in both the number and size of Aβ deposits at 4 and 6 months.

Reduced Aβ deposition findings align with several studies that crossed AD with α‐syn mouse models and reported a reduction in Aβ plaque deposition [28, 29, 31]. One study closely relevant to our work demonstrated that intracerebral injection of A53T α‐syn preformed fibrils in APP/PS1 mice led to a significant reduction in Aβ plaque deposition. This finding is noteworthy as it applied the same A53T mutation used in our model [56].

In contrast, only two studies have reported increased Aβ plaque deposition in AD and α‐syn mixed mouse models. One study observed a slight enhanced plaque accumulation in a coexpression model while another reported elevated Aβ deposition in 3xTg‐AD mice [30]. However, the latter model also expresses mutant human tau, which makes it difficult to isolate the specific effects of α‐syn on Aβ pathology. In contrast, our study and several other studies used AD mouse models [25, 28–31, 56] without mutant human tau expression, allowing a more focused assessment of how α‐syn influences Aβ pathology. Supporting this, studies have demonstrated that knocking out α‐syn in APP mouse models results in a significant increase in amyloid plaque burden [27, 28], further highlighting the modulatory effect of α‐syn on amyloid pathology.

Despite the absence of detectable α‐syn aggregation or direct evidence of Aβ/α‐syn coaggregation, the bigenic mice exhibited a reduction in both the number and size of Aβ deposits at 4 and 6 months. In addition, we observed differences in microglial morphology in the vicinity of Aβ deposits, suggesting that α‐syn expression may also influence local neuroinflammatory responses during early amyloid pathology.

Microglial phenotypes appear highly context dependent across neurodegenerative disorders, and microglia may play distinct roles at different stages and in different pathological contexts [57]. Consistent with this heterogeneity, postmortem studies in mixed pathologies have reported a relative lack of microglial activation compared with the activated phenotype observed in AD alone, potentially reflecting differences in neuropathological load and the inflammatory stimuli present [58]. In line with these human observations, we find that microglia in our mixed Aβ/α‐syn model retain a more ramified morphology at 4 and 6 months compared with the Aβ‐only model, suggesting that α‐syn expression may modulate microglial engagement with early amyloid pathology. Notably, similar region‐dependent differences in microglial morphology have been reported in human studies of mixed proteinopathies. For example, Bathe et al. observed increased numbers of P2RY12‐positive microglia with a highly ramified, homoeostatic morphology in certain cortical regions of cases with Lewy body or mixed pathology compared with AD alone, indicating that the presence of α‐syn pathology may influence the local microglial response [59]. However, future studies assessing aggregate conformation, oligomer burden and seeding capacity will be necessary to determine whether α‐syn primarily affects nucleation, elongation or conformational selection during Aβ assembly.

At the same time, several limitations should be considered when interpreting these findings. The analysis was performed at relatively early disease stages, when both amyloid deposition and inflammatory responses remain modest. In addition, the assessment of microglial responses relied primarily on morphological parameters. Variability in background staining between sections, likely related to differences in tissue processing and immunostaining performed in small batches, may also have influenced some of the quantitative measurements. Therefore, although the observed differences suggest that α‐syn expression may modulate early amyloid deposition and associated microglial responses, these findings should be interpreted with caution and will require further validation using complementary approaches and additional time points.

In conclusion, we observed that α‐syn expression is associated with altered early amyloid deposition and microglial morphology in vivo. In the Aβ/α‐syn model, Aβ deposits were fewer and smaller at early stages compared with Aβ‐only mice, while microglia surrounding Aβ microdeposits retained a more ramified morphology. Because no overt α‐syn aggregation was detected at these ages, these findings suggest that α‐syn expression may influence early amyloid deposition and the local neuroinflammatory environment independently of α‐syn aggregation. Thus, the Aβ/α‐syn model provides a system to examine how α‐syn expression modifies early amyloid deposition and microglial responses prior to the onset of overt α‐syn pathology. Future longitudinal and structural studies will be required to determine how α‐syn influences amyloid assembly, plaque architecture and downstream neuroimmune signalling during amyloid pathology progression.

## Author Contributions

Experiments were performed by Radhika Thakore, Markus Aldén, Valeriia Skoryk, Lukas Danielson and Lívia Lins. Methodology: Radhika Thakore, Valeriia Skoryk, Iran Augusto Neves da Silva, Markus Aldén, Nadja Gustavsson, Lukas Danielson, Lívia Lins, Jorge Domínguez Sánchez and Ana Rosenthal Arensburg. Formal analysis, visualisation and writing of the original draft: Radhika Thakore. Scientific supervision and manuscript review: Agnes Paulus, Sabine C. Konings, Andreas Heuer, Gunnar K. Gouras, Tomas Deierborg and Oxana Klementieva. Conceptualisation, resources and funding acquisition: Rakez Kayed and Oxana Klementieva.

## Funding

This work was supported specifically by the European Union’s Horizon 2020 Research and Innovation Program under the Marie Skłodowska‐Curie grant agreement no. 945378 (Generation Nano project, R. Thakore) and the Swedish Research Council (grant no. 2021–03149).

## Disclosure

This research was conducted as part of a PhD thesis in neuroscience at the Faculty of Medicine, Lund University, Sweden.

## Conflicts of Interest

The authors declare no conflicts of interest.

## Supporting Information

Additional supporting information can be found online in the Supporting Information section.

## Supporting information


**Supporting Information** Supporting Figure S1: Aβ accumulates, showing changes in size and morphology. Supporting Figure S2: Quantification of cortical Aβ microdeposits at early stages. Supporting Figure S3. Aβ accumulation at 2 months. Supporting Figure S4: Lack of cortical α‐syn at 4 months. Supporting Figure S5: Age‐dependent α‐syn (SNCA) levels in α‐syn and Aβ/α‐syn mice. Supporting Figure S6: Soluble Aβ42 levels in Aβ/α‐syn mice.

## Data Availability

The data that support the findings of this study are available upon request.
