# Supplementary material for: Early Amyloid Formation and Neuroinflammatory Response in a Bigenic Mouse Model Expressing Human α‐Synuclein and Aβ
Source: Parkinsons Dis. 2026 Jun 5;2026:7303965. doi: 10.1155/padi/7303965 (PMC13238505; doi:10.1155/padi/7303965)
Supplement: Supplementary file 1 — Supporting Information Supporting Figure S1: Aβ accumulates, showing changes in size and morphology. Supporting Figure S2: Quantification of cortical Aβ microdeposits at early stages. Supporting Figure S3. Aβ accumulation at 2 months. Supporting Figure S4: Lack of cortical α‐syn at 4 months. Supporting Figure S5: Age‐dependent α‐syn (SNCA) levels in α‐syn and Aβ/α‐syn mice. Supporting Figure S6: Soluble Aβ42 levels in Aβ/α‐syn mice. [file PADI-2026-7303965-s001.docx]

**Early amyloid formation and neuroinflammatory response in a bigenic mouse model expressing human α‑synuclein and Aβ**

**Supplementary Information**

Radhika Thakore^a^, Markus Aldén^f^, Nadja Gustavsson^a^, Lukas Danielson^a^, Lívia Lins^c,e^, Jorge Domínguez Sánchez^a^, Ana Rosenthal Arensburg^a^, Agnes Paulus^a^, Iran Augusto Neves da Silva^a^, Valeriia Skoryk^a^, Rakez Kayed^g^, Gunnar K. Gouras^b^, Tomas Deierborg^d^, Andreas Heuer^c,^, Sabine C. Konings^a,h^***** and Oxana Klementieva^a^*

**^a^**Medical Microspectroscopy, Department of Experimental Medical Science, NanoLund, Multipark, Lund University, Lund, Sweden.

**^b^**Experimental Dementia Research Unit, Department of Experimental Medical Science, Lund University, Lund, Sweden.

**^c^**Behavioural Neuroscience Laboratory, Department of Experimental Medical Science, Lund University, Lund, Sweden.

**^d^**Experimental Neuroinflammation Laboratory, Department of Experimental Medical Science, Lund University, Lund, Sweden.

**^e^**Neurophysiology Laboratory, Department of Physiology, Federal University of Sergipe, São Cristóvão, Brazil.

**^f^**Basal ganglia pathophysiology, Department of Experimental Medical Science, Lund University, Lund, Sweden.

**^g^**Department of Neurology and Mitchell Center for Neurodegenerative Diseases, University of Texas Medical Branch, TX, USA.

**^h^**Department of Molecular and Cellular Neurobiology CNCR, Center for Neurogenomics & Cognitive Research, Vrije Universiteit Amsterdam, Netherlands.

*** Corresponding authors:**

S. C. Konings: s.c.konings@vu.nl

O. Klementieva: oxana.klementieva@med.lu.se


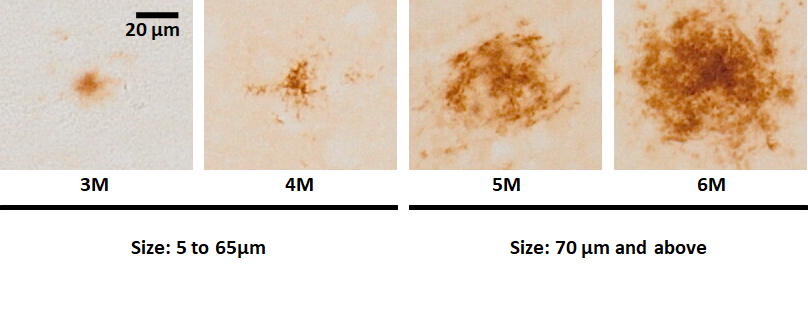


**Supplementary Figure S1: Morphology and size of Aβ aggregates.** Representative Aβ aggregates labeled with 82E1 found in brain tissue of Aβ/α-syn animals at various age points**,**


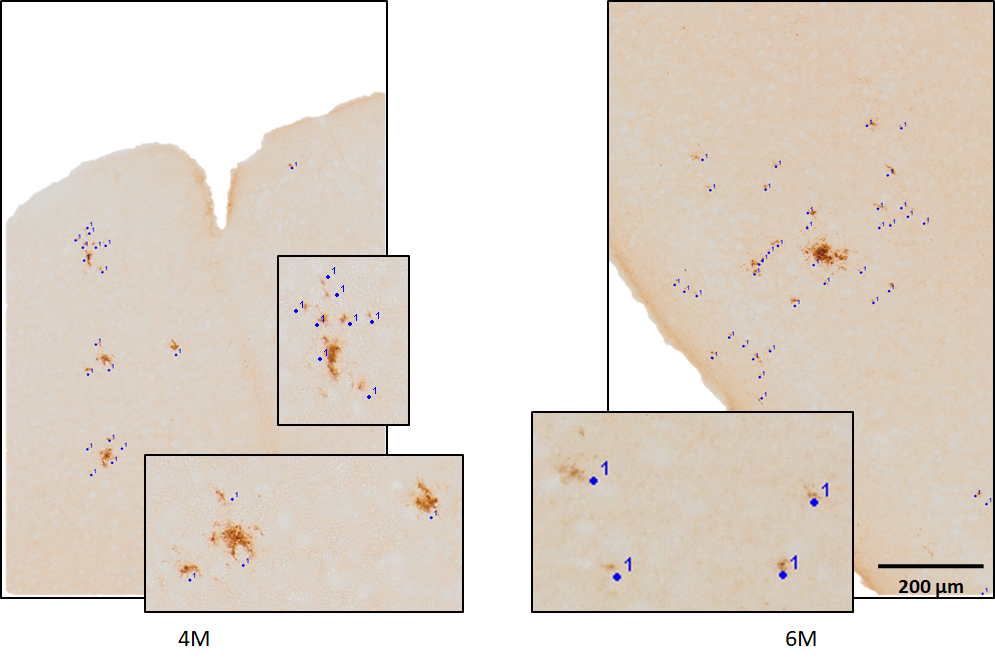


**Supplementary Figure S2: Quantification of cortical Aβ microaggregates at early stages.**
Representative brightfield images of cortical sections from 4-month (4M) and 6-month (6M) mice immunostained for Aβ (82E1). Insets show higher-magnification views of selected regions containing Aβ-positive deposits. Blue markers indicate individual microaggregates identified and included in the quantification. Microaggregates were defined as discrete, 82E1-positive deposits clearly distinguishable from background staining and separated from neighboring deposits. Whole cortical sections were imaged under identical acquisition settings. The Aβ microdeposits were identified manually based on signal distinguishable from the DAB background. It might therefore be better to remove the phrase “exceeding a predefined size and intensity threshold,” as it could be misleading.

.


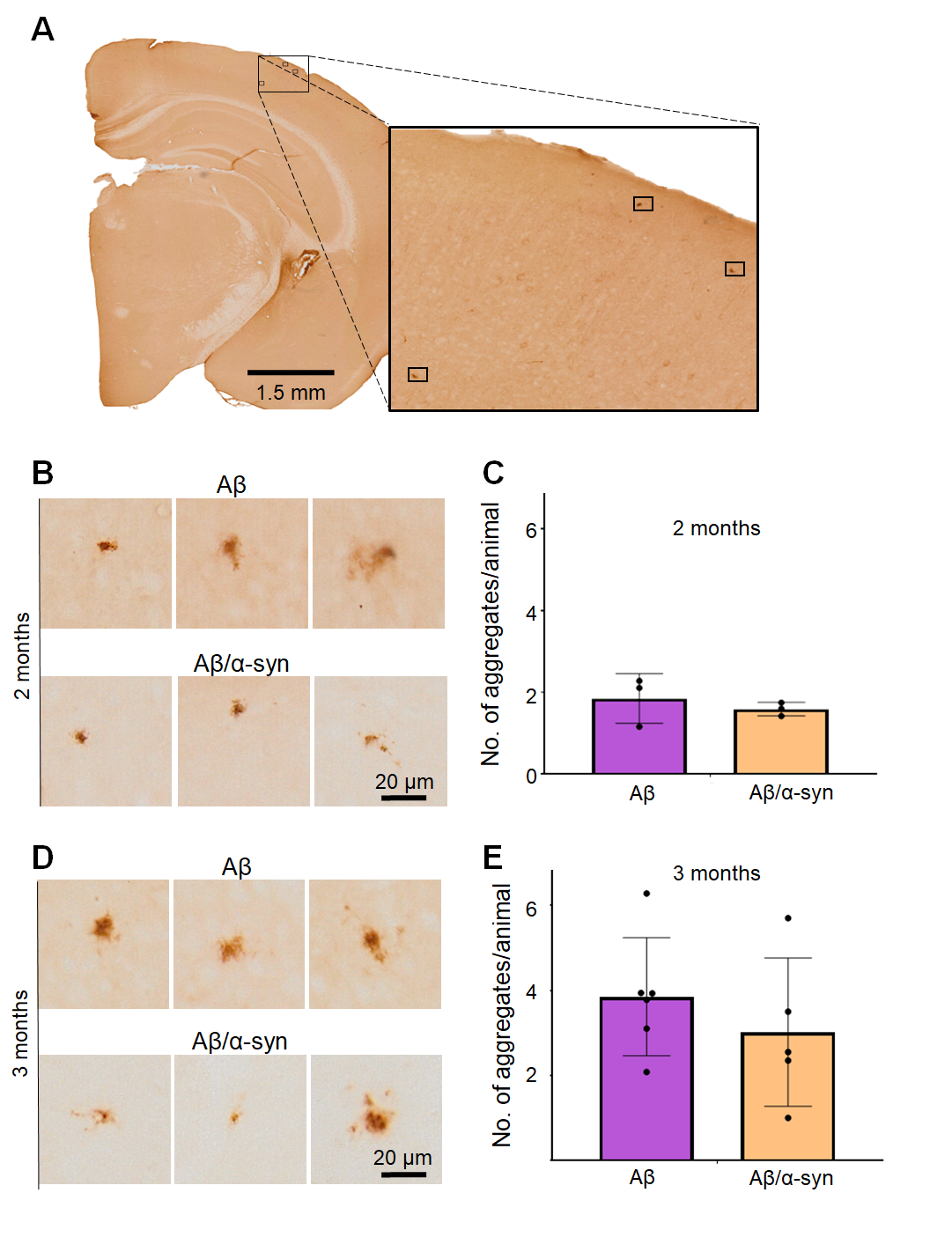


**Supplementary Figure S3. Aβ microdeposits at 2 and 3 months of age.** Representative images of cortical sections from Aβ and Aβ/α-syn mice immunolabelled with the 82E1 antibody against Aβ. Early Aβ microdeposits are detectable at both time points. No significant differences in the number of Aβ microdeposits were observed between genotypes at these ages. (**A**) Representative brain slice image of Aβ-only model showing size of 82E1-positive Aβ aggregates. (**B**) Morphology and size. of aggregates in Aβ-only and Aβ/α-syn mice at 2 months. (**C**) Quantification of the number of Aβ deposits per animal. Statistics: Mean ± SD, n=3 animals per genotype, a black dot represent one animal, Mann–Whitney U test, U=3, p=0.70. (**D**) Morphology and size. of aggregates in Aβ-only and Aβ/α-syn mice at 3 months. (**E**) Quantification of the number of Aβ deposits per animal. Statistics: Mean ± SD, n=5-6 animals per genotype, each black dot represents one animal, Mann Whitney U test, U=9, p=0.32.


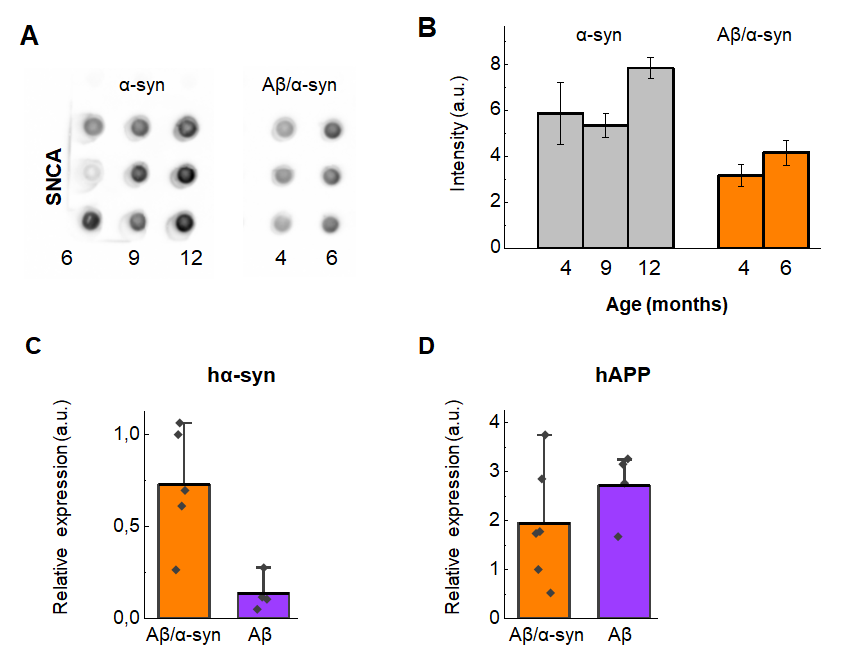
**Supplementary Figure S4: Age-dependent α-synuclein (SNCA) levels in α-syn and Aβ/α-syn mice**. (**A**) Representative dot blot analysis of α-synuclein protein levels in brain lysates from α-syn mice at 6, 9, and 12 months of age and from Aβ/α-syn bigenic mice at 4 and 6 months as detected by MABN389 antibody (SNCA). Each dot represents an individual animal. (**B**) Quantification of dot blot signal intensity expressed in arbitrary units (a.u.). In α-syn mice, α-synuclein levels increase with age, with the highest levels observed at 12 months. In Aβ/α-syn mice, α-synuclein is detectable at 4 and 6 months, with a modest increase between these time points. Equal amounts of total protein were loaded per spot as determined by BCA assay prior to membrane application. Signal intensities were quantified by densitometric analysis and normalized within each blot. Data are presented as mean ± SD Ages are indicated below each group.(**C**) Relative expression of human APP (hAPP) measured by qPCR in Aβ/α-syn and Aβ mice. (**D**) Relative expression of human α-synuclein (hSNCA) measured by qPCR in Aβ/α-syn and Aβ mice. Expression values were normalized to one reference animal and are presented as relative expression (a.u.). Bars represent mean ± SD, and individual data points correspond to biological replicates. Bars represent mean ± SD, with individual data points representing biological replicates.


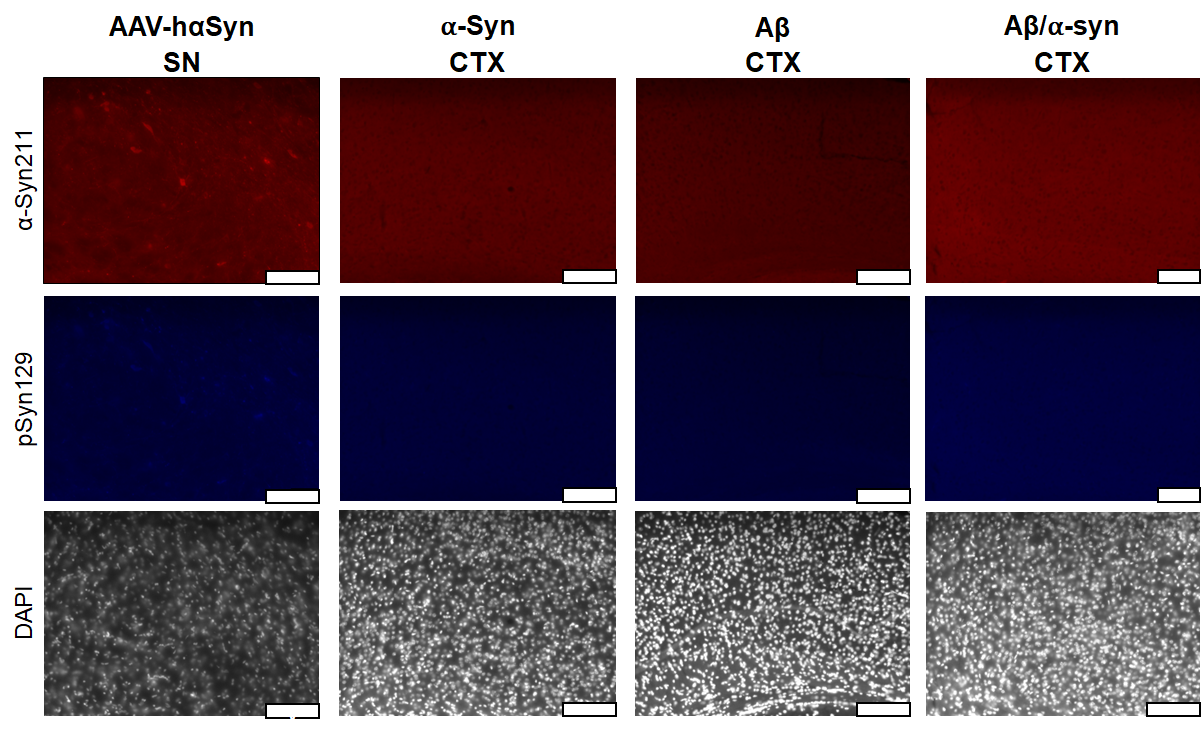
**Supplementary Figure S5: Lack of cortical α-synuclein at 4 months in Aβ-only and Aβ/α-syn mice.** Immunofluorescent representative images of brain tissue for total and phosphorylated α-synuclein in Substantia nigra (SN) of AAV-hαSyn (positive control) and cortex (CTX) sections from α-syn, Aβ, and Aβ/α-syn mice. DAPI counterstains nuclei. Scale bar is 100 μm.


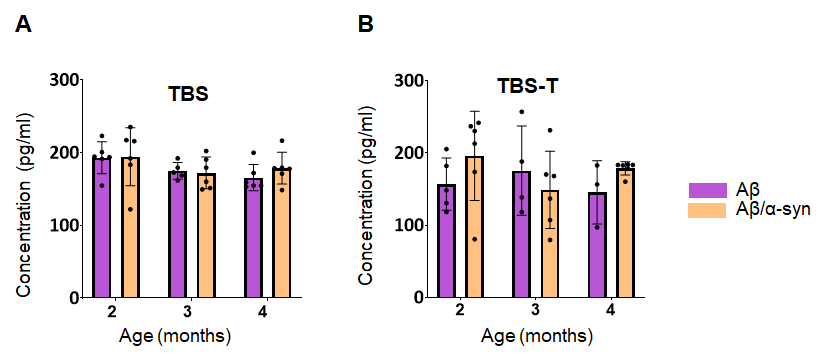


**Supplementary Figure S6: Mesoscale quantification of Aβ42 in soluble and membrane-**-**associated fractions of brain homogenate of Aβ and Aβ/α-syn mice.** (**A**) TBS, soluble, fraction of brain homogenate. Statistics: Mean ± SD, n=4-5 animals per genotype, each black dot represents one animal, two-way ANOVA, interaction, F_(2,29)_=0.32, p=0.72, age F_(2,29)_=2.86, p=0.07, genotype F_(1,29)_=0.21, p=0.64. (**B**) TBS-T, membrane-associated, fraction of brain homogenate. Statistics: Mean ± SD, n=4-5 animals per genotype, each black dot represents one animal, two-way ANOVA, interaction, F_(2,24)_=1.41, p=0.26, age F_(2,24)_.

**Brain Homogenization for Aβ42 quantification**

Right hemispheres were homogenized in TBS (1:10 w/v) supplemented with 1% Halt™ Protease and Phosphatase Inhibitor Cocktails (Thermo Fisher Scientific, 78430, 78428) using Lysing Matrix D beads (FastPrep-24™ 5G) and a TissueLyser II (Qiagen; 27 Hz, 1 min, 4°C). Homogenates were centrifuged (14,000 × g, 30 min, 4°C), and the supernatant collected as the soluble (TBS) fraction and snap-frozen.

The pellet was re-suspended in TX-TBS (1% Triton X-100, with inhibitors), incubated on ice (30 min), and centrifuged again (14,000 × g, 30 min, 4°C). The supernatant was collected as the membrane-soluble (TX-TBS) fraction.

All fractions were stored at –80°C. Aβ42 levels in TBS and TX-TBS fractions were quantified using the MSD V-PLEX Human Aβ42 Peptide (6E10) Kit (K15200E, Mesoscale) according to manufacturer’s instructions. The samples were analyzed in single replicates.
